# Feasibility and utility of mapping disease risk at the neighbourhood level within a Canadian public health unit: an ecological study

**DOI:** 10.1186/1476-072X-9-21

**Published:** 2010-05-10

**Authors:** Eric J Holowaty, Todd A Norwood, Susitha Wanigaratne, Juanjo J Abellan, Linda Beale

**Affiliations:** 1 Population Studies and Surveillance, Cancer Care Ontario, 620 University Avenue, Toronto, Ontario, Canada; 2 Dalla Lana School of Public Health, University of Toronto, 155 College St., Toronto, Ontario, Canada; 3 CIBER Epidemiología y Salud Pública (CIBERESP) and Centre for Public Health Research (CSISP), Valencia, Spain; 4 Small Area Health Statistics Unit, MRC-HPA Centre for Environment and Health, Imperial College London, UK

## Abstract

**Background:**

We conducted spatial analyses to determine the geographic variation of cancer at the neighbourhood level (dissemination areas or DAs) within the area of a single Ontario public health unit, Wellington-Dufferin-Guelph, covering a population of 238,326 inhabitants. Cancer incidence data between 1999 and 2003 were obtained from the Ontario Cancer Registry and were geocoded down to the level of DA using the enhanced Postal Code Conversion File. The 2001 Census of Canada provided information on the size and age-sex structure of the population at the DA level, in addition to information about selected census covariates, such as average neighbourhood income.

**Results:**

Age standardized incidence ratios for cancer and the prevalence of census covariates were calculated for each of 331 dissemination areas in Wellington-Dufferin-Guelph. The standardized incidence ratios (SIR) for cancer varied dramatically across the dissemination areas. However, application of the Moran's I statistic, a popular index of spatial autocorrelation, suggested significant spatial patterns for only two cancers, lung and prostate, both in males (p < 0.001 and p = 0.002, respectively). Employing Bayesian hierarchical models, areas in the urban core of the City of Guelph had significantly higher SIRs for male lung cancer than the remainder of Wellington-Dufferin-Guelph; and, neighbourhoods in the urban and surrounding rural areas of Orangeville exhibited significantly higher SIRs for prostate cancer. After adjustment for age and spatial dependence, average household income attenuated much of the spatial pattern of lung cancer, but not of prostate cancer.

**Conclusion:**

This paper demonstrates the feasibility and utility of a systematic approach to identifying neighbourhoods, within the area served by a public health unit, that have significantly higher risks of cancer. This exploratory, ecologic study suggests several hypotheses for these spatial patterns that warrant further investigations. To the best of our knowledge, this is the first Canadian study published in the peer-reviewed literature estimating the risk of relatively rare public health outcomes at a very small areal level, namely dissemination areas.

## Background

Interest in mapping and spatial analysis of disease burden and healthcare utilization has increased substantially over the past two decades [1-5]. Within local health authorities (e.g., public health units or PHUs), this interest has shifted from large to small area analyses, in keeping with emerging responsibilities in neighbourhood-based planning and environmental risk assessment [6-8]. With the increasing availability of data on health and population characteristics, as well as on environmental hazards and behavioural risk factors and other determinants of ill-health, PHUs and other agencies now have access to huge georeferenced data holdings [9-11]. Advances in computing power and the availability of sophisticated mapping software [12-14], as well as the public's keen interest in the effects of environmental pollution, add urgency to the use of geographic information systems in public health [15-19]. The recent introduction of the Ontario Public Health Standards has reinforced the importance of PHUs using a "determinants of health" approach to identifying risk at the local level [6]. For PHUs, this equates to the examination and understanding of health status variation across small area geographies to tailor public health interventions, address inequities, reduce risk, and better meet the needs of priority populations. Health units in Ontario need powerful analytical tools to meet the growing surveillance requirements of the new foundational standards. This paper focuses on cancer incidence mapping within one PHU area.

Disease mapping is often carried out to visualize and explore spatial variations in risk. This may generate new causal hypotheses, perhaps to provide an important context for future analytic studies, or it may support program planning and evaluation. Generally, several goals may be important for mapping disease risk using choropleth maps: 1) to accurately estimate the rate or risk within each area; 2) to discover spatial patterns or clusters in the data, whether for unusually high or low rates; and 3) to compare the pattern between maps [20-22]. It is important to note that no single approach is generally optimal for all goals; thus, prioritization may be important in planning the spatial analysis [21,22]. Here, we define a cluster as one or more contiguous areas (dissemination areas or DAs) having an excess of cases indicated by elevated standardized incidence ratios (SIR) and relatively high probabilities of exceeding background estimates of risk, as determined from a hierarchical Bayesian model [3,23]. It is the aim of this paper to illustrate the feasibility and utility of generating informative maps of the spatial pattern of public health problems at the small area level, within a single PHU area, using various types of cancer as examples.

## Methods

### Data Sources

Four data types are typically required to conduct geographic analysis of disease risk: (1) health outcome data; (2) potentially explanatory covariate data; (3) geographic boundary files; and (4) population data. A description of these four data types follows.

#### Incident cancers

All incident cancers from 1999 through to 2003, used as numerator data, were obtained from The Ontario Cancer Registry (OCR), administered by Cancer Care Ontario. The OCR is a passive registry that covers the entire province, capturing all new cases of invasive neoplasia, except for non-melanoma skin cancers. In terms of completeness of case ascertainment, an independent field study conducted in 2002 estimated that completeness of histologically confirmed cases was 98.5% [24]. Completeness of the 6-digit postal code describing residence at the time of diagnosis was 97.9%, for cases incident over the interval 1999-2003. It should be noted that information on race and ethnicity are absent from the OCR, and stage at diagnosis was only captured for a minority of registered cases over the period of inquiry. Further details about the operation of the OCR, and data quality, can be found in a recent monograph [25]. First and later primary cancers were included in the analyses described in this paper.

Geocoding of the incident cancer file was done using a postal code conversion file (PCCF+ Version 4J) [26]. This conversion file assigns a full range of geographic identifiers, based on the 2001 Canadian Census. Statistics Canada classifies Canadian geography using two systems; the Standard Geographic Classification (SGC) [27] and the Statistical Area Classification (SAC) [28]. The SGC is a hierarchical classification that breaks down provinces and territories into census divisions (CDs), CDs into census subdivisions (CSDs), and CSDs into DAs. DAs are the smallest geographic unit at which Statistics Canada reports complete census information and typically consists of between 400 and 700 people [29]. The SAC is also used for data dissemination purposes and breaks down urbanized areas of Canada into census metropolitan areas (CMAs), census agglomeration areas (CAs), census tracts (CTs) and DAs. For valid postal codes, PCCF+ can assign geographic codes accurately [26]. Where postal codes serve more than one DA, which occurs in both rural and urban areas of Canada, postal codes are assigned to DAs based on an unbiased, population weighted random allocation method. In cases where valid postal codes cannot be used to assign the full range of geographic identifiers, the first two or three characters in the postal code are used to assign partial geography.

PCCF+ also assigns the *Neighbourhood Income Quintile *(QAIPPE) [26]. The Neighbourhood Income Quintile used in this study is based on the average 2000 income per single-person equivalent for 2001 DAs. The quintile value assigned to a given DA is based on the distribution of average household income values of DAs that fall within the local census CMA or CA, or among those DAs that fall outside the boundaries of any CMA/CAs [30].

Cancer sites were selected for the four most common incident cancers and those sites for which statistically significant spatial aggregation was reported in recent Canadian cancer atlases (Additional file 1 - Table S1 lists the sites) [31,32]. For each site, the indirectly adjusted SIRs and 95% confidence intervals were calculated for the total study area (Wellington-Dufferin-Guelph or hereafter, WDG), using DA-coded case data and all Ontario as the comparator [33]. Since the Besag-York-Mollié (BYM) model [13] (described in Additional file 2 - Appendix A), is time consuming to apply to many sites, we first utilized Moran's *I *to test the spatial distribution of the observed SIR values for spatial autocorrelation (Additional file 1 - Table S1) [34-37]. Those sites with statistically significant one-tailed p-values (p < 0.002, adjusted for 24 tests using Bonferroni correction) and positive values of Moran's *I *were then analyzed with the BYM model to better estimate areal rates and visualize spatial patterns.

#### Geographic Boundary Files

The geographic boundary files used for analysis in this paper are electronic map layers based on each of the hierarchical divisions in the SGC and SAC classifications described above. These boundary files were obtained from the Public Health Agency of Canada [38] and were formatted for use within the Rapid Inquiry Facility (RIF) (see data processing below) and WinBUGS (Bayesian inference Using Gibbs Sampling, running under Microsoft Windows) [39,40] to characterize and visualize the areas of interest.

#### Populations

Ontario population counts, stratified by five-year age groups (0 to 85+), sex, and various geographic areas described above, were obtained from the Statistics Canada 2001 Census. These Ontario population data were used to calculate indirectly standardized rate ratios.

### Data Processing

The RIF Version 3.12, developed by the Small Area Health Statistics Unit at Imperial College London, is an extension to ArcGIS Desktop [12]. Designed for spatial surveillance through the creation of disease maps, and for assessment of health risks related to environmental hazards, the RIF uses open database connectivity to calculate directly and indirectly standardized rates and rate ratios by user-selected geographic areas [41]. The geographic areas available for our analyses were defined during the creation of the RIF database, which may be in either Microsoft Access or Oracle. Additionally, the RIF interfaces with WinBUGS and SaTScan to provide Bayesian hierarchical smoothing and cluster identification, respectively [39,42]. The SIRs by 2001 DA for the WDG study area were calculated using the RIF [41], and BYM spatial smoothing was performed using the RIF interface with WinBUGS. Final map production was performed using ArcGIS Desktop version 9.3 [43]. In testing for spatial clustering, our null hypothesis was that the SIRs at the DA level were independent; our alternate hypothesis was that the SIRs at the DA level were clustered, where spatial covariance was stationary throughout the study area.

Moran's *I *was calculated for the distribution of SIRs to assess the overall spatial correlation between neighbouring DAs, while adjusting for population density [35-37]. Moran's *I *ranges from -1 to +1, with a positive/negative sign representing positive/negative spatial autocorrelation and zero representing no spatial autocorrelation [44]. While testing for spatial autocorrelation is typically performed with the asymptotic normal distribution of Moran's *I *test statistic, this assumption of normality is often not satisfied [45]. Thus, we implemented a parametric bootstrap Moran's *I *from the DCluster package in R using the observed and expected counts for each DA [36]. Sample code is provided in Additional file 3 (Appendix B). This implementation of Moran's *I *simulated the observed values for each DA, based on the Poisson distribution, to calculate the likelihood of the observed pattern occurring randomly. Thus, Moran's *I *provides both a test of significance and measure of the strength of clustering or dispersion. First-order neighbours were used for consistency with the definition of neighbours used in the BYM model and were favoured over distance-based neighbours given the large variation in geographic size of the DAs resulting from the mix of rural and urban environments in the study area.

### Estimation of Small Area Relative Risk

The observed count *Y*_*i *_of incident cases in area *i *is influenced by the age- and sex-adjusted expected count *E*_*i *_and the area's relative risk, *θ*_*i*_. The RIF computes the *E*_*i *_from the vector *P*_*i *_of age- and sex-specific population counts of area *i*, and a vector of estimated rates, *ψ *for each of these groups, with *E*_*i *_= *P*_*i*_'*ψ*. The age- and sex-specific rates *ψ *are estimated using the Ontario-wide dataset for the same time period. Within the RIF, when an explanatory variable is included (e.g., average household income), this variable must be categorical (quintiles in this paper) and the incidence rate is calculated for each age/sex/income-quintile group. The *E*_*i *_are then calculated as above.

As the number of cases in an area is often small and the outcome rare, the case counts are modeled with a Poisson distribution with

the maximum likelihood estimate of *θ*_*i *_is known as the Standardized Incidence Ratio, *θ*_*i *_= *Y*_*i*_/*E*_*i*_. However, the small counts for the *Y*_*i *_typically make the SIRs unreliable with a large proportion of zeros; often, the most extreme risks present in small regions with low *E*_*i *_and one observed incident case. To overcome this problem, a spatial random effects model is specified.

The simplest and most commonly used spatial model for disease mapping applications is the BYM model, which models the risk in a hierarchical Bayesian fashion, incorporating appropriate random effects terms for both spatially correlated and uncorrelated random errors [13,15,23,46-48]. Further details about the BYM model we employed is described in Additional file 2 (Appendix A), along with explanatory notes. This model is in a form that supports Bayesian inference with the software package WinBUGS, which uses a Markov chain Monte Carlo algorithm with Gibbs sampling [39,40]. Model fitting was carried out using three separate chains starting from different initial values. Convergence was checked by visual inspection of time series plots of samples of: SIRs; autocorrelations; standard deviations of the spatial and non-spatial random effects; and by computing the Gelman-Rubin diagnostic [49,50]. The first 100,000 samples from each chain were discarded as burn-in. Then, each chain was run for a further 1,000,000 iterations, with every 200^th ^sample saved, yielding a total simulation of 3,000,000 iterations and 15,000 simulations to summarize.

The predicted SIRs were calculated as the means of the posterior distributions for each area, along with their 95% credible intervals; the probability of each area having above-average risk (i.e., SIR>1.0) was also computed. The fraction of the variation in risk that could be attributed to the spatial component was also estimated along with a 95% credible interval. We utilized elliptical analysis with the spatial scan statistic (SaTScan) to corroborate findings from the BYM model, with the intent to show results from a commonly used package for spatial analysis with which readers are likely familiar [42]. The added value of the BYM model lies in the ability to map the smoothed rates to display underlying spatial patterns that may exist in the data.

### Presentation of Results

Since colour scheme influences pattern recognition and map readability, and diverging and sequential colour schemes provide better recognition of clustering, we used a red-grey scheme, where red hues represent higher rates, grey shades represent lower rates, and white represents rates comparable to the reference population's rates [51,52]. We overlaid a layer of cross-hatching to indicate the statistical significance of the SIR estimates, an improvement to basic choropleth mapping [52].

While there is some evidence that quantile classifications provide more accurate interpretation of individual values and improve pattern recognition, we used consistent legend class intervals to facilitate between map comparisons [52,53]. Seven classes are used in our choropleth maps - the recommended maximum number of classes [52]. As we are mapping SIRs, a natural class centred about 1.0, a diverging colour scheme that divides risks into high and low classes is suitable. Class break points were selected by examining the distribution of the raw and smoothed SIRs and rounding the break points to intuitive levels.

**Figure 1:**
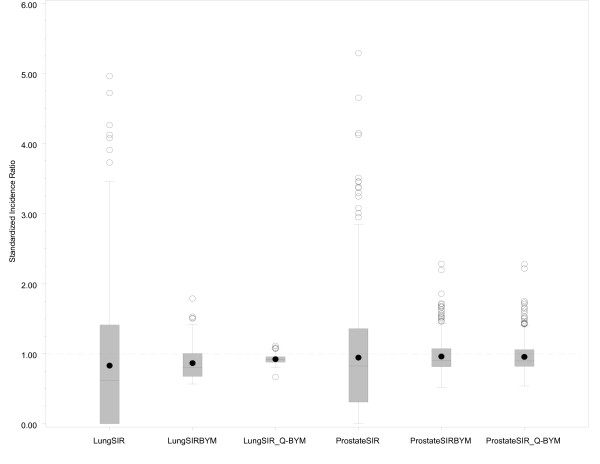
**Box plot of SIR variation at the DA level for male lung cancer and prostate cancer raw SIRs, BYM smoothed SIRs and household income adjusted, BYM smoothed SIRs**. The length of the rectangular box represents the interquartile range (25^th ^percentile to the 75^th ^percentile), the line in the box represents the median value, the filled circles within the box represent the mean value, the lower whisker extends to the first quartile minus 1.5 times the interquartile range, the upper whisker extends to the upper quartile plus 1.5 times the interquartile range, the unfilled circles represent those data points that are beyond the upper whisker, and the dashed line at 1.0 represents the provincial average. The y-axis scale was reduced to improve the visibility of the box plot, and resulted in the removal of three extreme values from LungSIR and one extreme value from ProstateSIR.

A box-plot (Figure 1), is used to display the distribution of SIRs at the DA level and the shrinkage in these estimates through fitting the BYM model.

## Results

**Figure 2:**
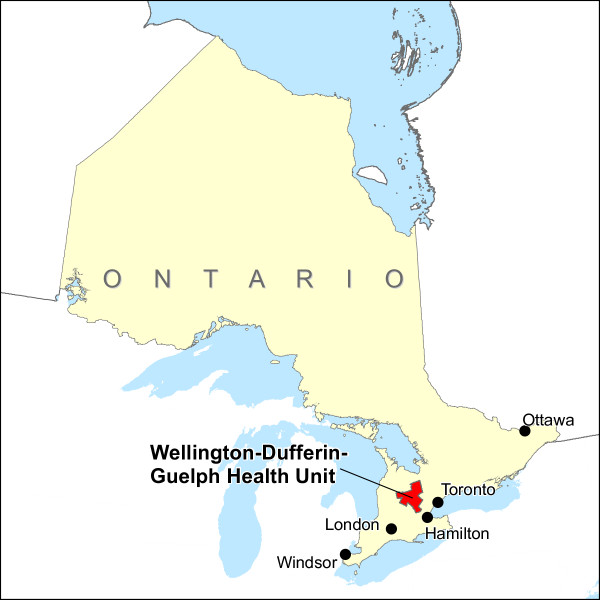
**Wellington-Dufferin-Guelph Health Unit location**.

The area served by WDG Public Health is displayed in Figure 2. It comprises 0.5% of the land area of Ontario, and 2.1% of the population, as of the 2001 Census of Canada (see Additional file 4 - Table S2). In terms of land use, approximately 2.9% of WDG land area is designated as urban (containing 61.5% of the WDG population), and 97.1% as rural (containing 38.5% of the WDG population) [54].

The City of Guelph and the Town of Orangeville are the most populous centres, comprising 45% and 11% of the WDG population respectively. These urban centres are located only 99 km and 84 km respectively from Toronto, Canada's largest municipality. The entire land area of WDG is divided into 331 DAs, of which all but 8 are populated or have sufficient age-sex data available from the 2001 Census. In terms of average annual household income, a larger proportion of the WDG population is in the highest income quintile relative to Ontario as a whole (see Additional file 4 - Table S2). Further, within WDG, the rural areas of Wellington County (excluding Guelph) and Dufferin County (excluding Orangeville) are characterized by a larger proportion of the population in the top two income quintiles relative to Guelph and Orangeville.

The frequency of select sites of incident cases diagnosed in WDG over the interval 1999-2003 is shown in Additional file 1 (Table S1). These sites were selected from recent atlases in which statistically significant spatial aggregation was reported at the census division level [31,32]. Not surprisingly, the number of incident cases occurring within each DA is low, with a median value of zero for all but the four commonest cancers. The SIR for all DAs combined within WDG is significantly different from unity only for female cutaneous melanoma (SIR = 1.33; 95% CL 1.10-1.61), relative to Ontario as a whole. In terms of spatial autocorrelation, the Moran's *I *statistic (bootstrapped) is significantly different from zero only for male lung (p < 0.001) and prostate cancer (p = 0.002). Moran's *I *provided a rapid assessment of those cancer sites which most likely exhibited distinct spatial patterns; thus, the computation-intensive BYM [13] models for disease mapping were reserved for those sites where clustering was most likely to be occurring.

**Figure 3:**
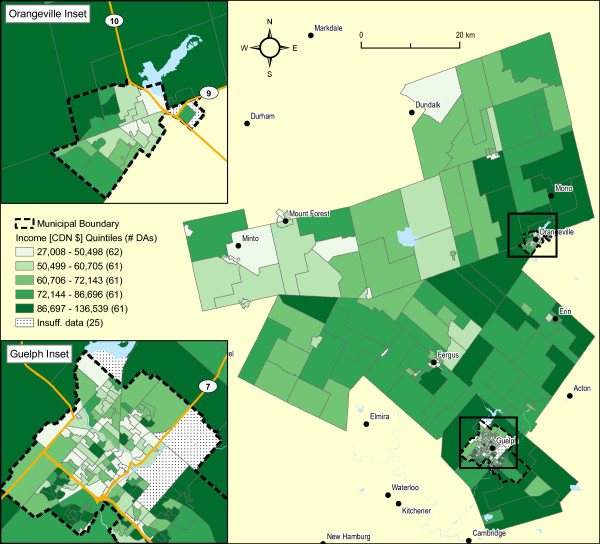
**2000 Average household income by 2001 DA, WDG**. Moran's I: 0.46 (p < 0.001).

Figure 3 is a map of average annual household income at the DA level for the year 2000. The Moran's *I *value of 0.46 (p < 0.001) is indicative of strong spatial association. Visually, average household income appears higher in the non-urban areas surrounding Guelph and Orangeville. This is confirmed in Additional file 4 (Table S2). This finding is important because of the strong association between household income (whether an individual or areal measure) and other measures of socio-economic status, and certain behavioural factors known to be associated with cancer risk (e.g., smoking, obesity, physical inactivity and screening behaviour) [55-57].

**Figure 4:**
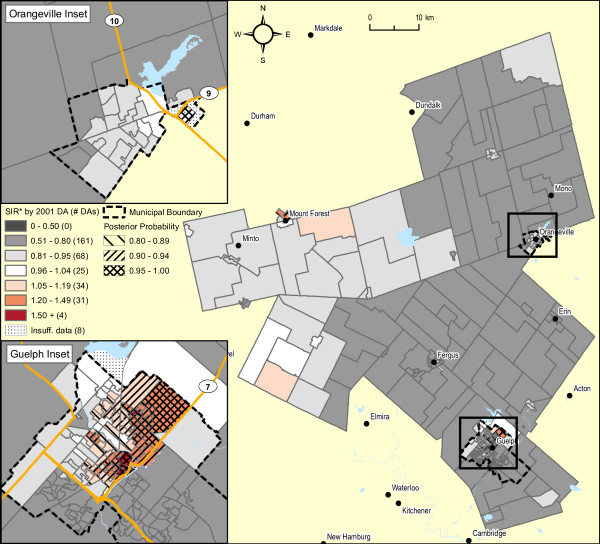
**Male lung cancer incidence 1999-2003 (337 observed cases), WDG, full Bayesian smoothing, by 2001 DA**. Total: 331 DAs; Overall SIR: 0.79 (95% CI: 0.70-0.90); WinBUGS fracspatial: 0.94 (95% CI: 0.44-1.00). Indirectly standardized incidence ratios calculated for all ages, using Ontario age-specific rates, 1999-2003. Full Bayesian smoothing using the BYM model [13]. Excludes 3.1% of male lung cancer cases with missing or invalid residential postal code at diagnosis.

Male lung cancer smoothed risk ratios are displayed in Figure 4. The original raw SIR map is not shown, both because of residual risk of disclosure and because the map is visually uninformative due to the instability of SIR estimates. The raw SIR estimates input to the fully hierarchical Bayesian BYM model have undergone substantial smoothing, illustrated in Figure 1, largely because of strong spatial autocorrelation. This is evidenced by the high value for the spatial fraction of the modeled random variation, 93.5% (95% CI 43.8 - 99.9%). In the northeast sector of Guelph, 63 of 94 DAs (67%) exhibit SIRs with posterior probabilities of 0.80 or higher (i.e., relative to the rate of lung cancer in Ontario males). Elliptical SaTScan [42] analysis of the original raw SIRs also confirms a cluster in this same sector of Guelph (figure not shown), including 67 contiguous DAs (SIR = 2.2, relative to the remainder of WDG, p = 0.001; SIR = 1.53 relative to Ontario, p < 0.001; Obs = 107 cases).

**Figure 5:**
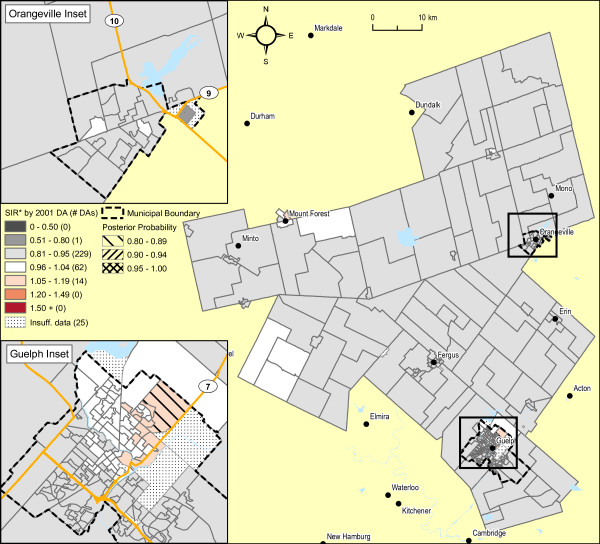
**Male lung cancer incidence 1999-2003 (333 observed cases), WDG, full Bayesian smoothing and adjusted for average household income quintiles, by 2001 DA**. Total: 331 DAs; Overall SIR: 0.91 (95% CI: 0.80-1.01); WinBUGS fracspatial: 0.61 (95% CI: 0.01-1.00). Indirectly standardized incidence ratios calculated for all ages, using Ontario age-specific rates, 1999-2003, adjusted for average household income quintiles (2001 Census DAs). Full Bayesian smoothing using the BYM model[13]. Excludes 3.1% of male lung cancer cases with missing or invalid residential postal code at diagnosis and 1.2% of cases due to suppressed income data for the 2001 Census.

Adjustment of these SIR estimates by average annual household income quintile shows considerable attenuation in the range of SIR values (see Figures 1 and 5), now with only one DA in the northeast sector of Guelph displaying an increased SIR with an exceedence probability of > = 0.80. Of relevance, the spatial pattern of SIRs for female lung cancer did not show any evidence of spatial structure association, whether unadjusted or adjusted for household income (not shown). Further, comparison of smoothed areal risk estimates of male oropharyngeal, laryngeal, esophageal, bladder and renal cancers with lung cancer failed to show any correlation, other than for upper aero-digestive cancers combined (Spearman's rank correlation coefficient, r = 0.48; p < 0.0001).

**Figure 6:**
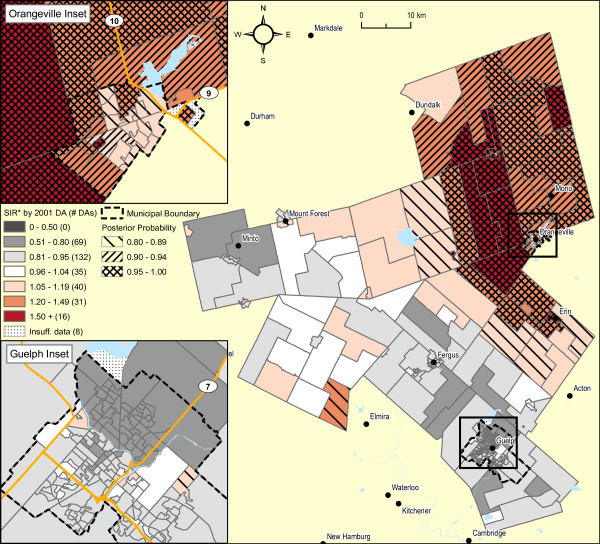
**Prostate cancer incidence 1999-2003 (735 observed cases), WDG, full Bayesian smoothing, by 2001 DA**. Total: 331 DAs; Overall SIR: 0.90 (95% CI: 0.82-0.98); WinBUGS fracspatial: 0.90 (95% CI: 0.46-1.00). Indirectly standardized incidence ratios calculated for all ages, using Ontario age-specific rates, 1999-2003. Full Bayesian smoothing using the BYM model[13]. Excludes 1.4% of prostate cancer cases with missing or invalid residential postal code at diagnosis.

Prostate cancer risk in males, output from the BYM model, is displayed in Figure 6. Similar to male lung cancer, the raw SIRs have undergone substantial smoothing, largely because of strong spatial autocorrelation (see Figure 1). In the Town of Orangeville, and its suburban and rural surroundings stretching north to Dundalk and south beyond Erin, 57 of 74 DAs (77%) are displaying higher SIRs with exceedence probabilities > = 0.80. Again, elliptical SaTScan analysis corroborates this finding, with detection of a statistically significant cluster of 58 contiguous DAs, in Orangeville and its surroundings (combined SIR = 1.9 relative to the remainder of WDG; p = 0.001; SIR = 1.53 relative to Ontario, p < 0.001; Obs = 227 cases).

**Figure 7:**
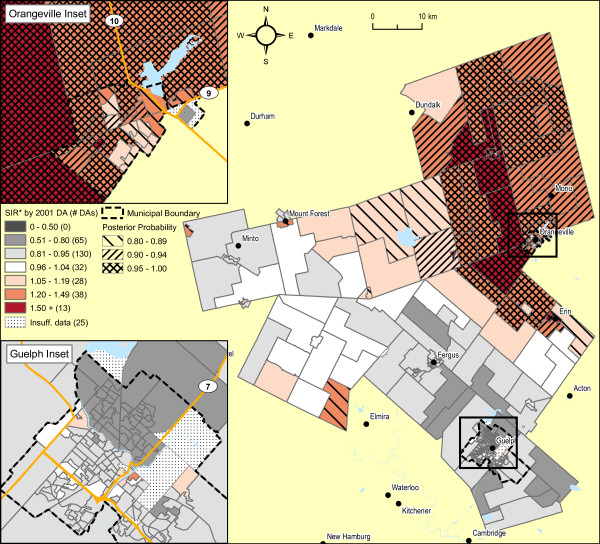
**Prostate cancer incidence 1999-2003 (723 observed cases), WDG, full Bayesian smoothing and adjusted for average household income quintiles, by 2001 DA**. Total: 331 DAs; Overall SIR: 0.90 (95% CI: 0.82-0.98); WinBUGS fracspatial: 0.92 (95% CI: 0.51-1.00). Indirectly standardized incidence ratios calculated for all ages, using Ontario age-specific rates, 1999-2003, adjusted for average household income quintiles (2001 Census DAs). Full Bayesian smoothing using the BYM model[13]. Excludes 1.4% of prostate cancer cases with missing or invalid residential postal code at diagnosis and 1.6% of cases due to suppressed income data for the 2001 Census.

Adjustment of these SIR estimates for prostate cancer by average household income quintile shows minimal attenuation in the range of SIR values, with a spatial pattern little changed from the original Bayesian analysis (see Figures 1 and 7).

## Discussion

Within WDG, it is noteworthy that a significantly higher risk of lung cancer (SIR = 1.53) exists in a substantial section of the City of Guelph, in spite of an overall deficit in risk across all WDG (SIR = 0.85). The dramatic attenuation in this spatial pattern as a result of adjustment for household income supports the hypothesis that higher rates of smoking, historically, may be the predominant causal factor, as opposed to local factors in the ambient environment. Unfortunately, smoking prevalence estimates collected in population sample surveys are unable to confirm this hypothesis, as they typically report only at the much larger PHU area level [58]. However, the similarity in spatial pattern for upper aero-digestive cancers (oropharynx, esophagus and larynx), also known to be associated with tobacco consumption [59], supports this hypothesis. The lack of a similar spatial pattern for female lung cancer may simply reflect the later evolution of tobacco consumption in females, and the long delay in appearance of associated solid tumours [60].

It should be noted that north Guelph had a strong manufacturing presence for many decades, with about 25% of the Guelph workforce employed in such manufacturing sectors as transportation, equipment, machinery and fabricated metal, wood, electrical and chemical production [61]. Of note, an iron foundry opened in 1912 and was in continuous operation up until 1989, when it abandoned its operation in north Guelph leaving behind extensive pollution on its 13 acre site [62]. Finally, the existence of a large cigarette manufacturing facility for nearly 50 years in north Guelph also adds credence to the hypothesis of a high prevalence of smoking among local residents, many of whom likely worked at this facility [63].

The data used from the cancer registry are somewhat dated, and it will be informative to compare our findings with more recent data, soon to be available for the period 2004-2008. Additionally, while we did examine spatial patterns of other tobacco-associated cancers in men, the associations with lung cancer were not consistent, possibly because of the multifactorial etiology of these other sites. Newer methods of joint pattern analysis, whether spatial or spatio-temporal, may permit partitioning the underlying risk surface into shared and disease-specific components, thereby providing more convincing evidence of real clustering [64,65]. Extension of Bayesian spatio-temporal models to joint spatio-temporal models will permit the comparison of patterns where the effects may lag more in one group than another, as we hypothesize for female lung cancer [60].

Our finding of a significantly higher risk of lung cancer in one part of Guelph demonstrates that the a priori identification of a local cluster, in the presence of plausible risk factor attribution, can serve as a useful basis for focused follow-up investigation and possible interventions (e.g., local smoking cessation services) [66,67].

Detection of a significantly higher risk of prostate cancer in the Town of Orangeville and its surroundings was an unexpected finding in our analysis (SIR = 1.53). In terms of potential explanatory factors, there are no known etiologic factors that have as strong an association as smoking and lung cancer. Prior to the prostate-specific antigen (PSA, a screening test for detection of prostate cancer) era, studies of the possible association between socioeconomic status (SES) and prostate cancer were inconsistent; however, since the introduction of PSA screening in the late 1980s, a positive correlation is now being reported more consistently, particularly in the U.S.A. [68-71]. Our adjustment of SIR estimates for neighbourhood income did not influence the spatial pattern much, perhaps because SES is not related to prostate cancer incidence in Ontario, or perhaps average household income quintile is not the ideal measure for SES. A plausible hypothesis would be that PSA testing of older men was more prevalent in the Orangeville area, leading to more timely detection of prostate cancer, in both clinically apparent and indolent forms. This is a common hypothesis held by others in explaining recent spatial patterns of prostate cancer [72-74]. Unfortunately, no information about PSA testing exists during this period of time to confirm this. While patients did not necessarily pay for PSA screening, it was an uninsured test over this period and payment largely depended on the type of laboratory processing the specimen [75]. It should be noted that the apparent size of this cluster of cases argues against local environmental sources as causal agents. There seems to be little difference in risk, whether men live in urban or rural areas.

Finally, it will be useful to monitor this cluster more closely, perhaps confirming initially its persistence in the more recent period, 2004-2008, once these cancer data are available. With the recent decision of the Government of Ontario to permit labs to bill the Provincial Health Plan for PSA screening, we may soon have georeferenced data on PSA testing and be able to adjust our analysis accordingly [76]. Finally, in order to sort out whether this excess is mostly attributed to the more indolent form of prostate cancer, spatial analysis using georeferenced mortality data for prostate cancer would be useful, given that stage at diagnosis was incomplete for cases registered in the OCR up to 2006. From 2007 and on, though, efforts are now being made to capture stage with sufficient completeness to support population studies.

DAs, CTs and other small administrative or statistical areas have traditionally been viewed as less than optimal for spatial analysis, because of their variability in size (geographic and demographic), and inconsistency over time. CT coverage is incomplete outside urban areas. For example, in Ontario, approximately 20% of the population, living across 96.6% of the land area of Ontario, are not included in CTs [6]. For DAs, population coverage is complete, but census data are suppressed or rounded because of confidentiality concerns. In the Canadian census, suppression of data at the DA level occurs if the DA has less than 250 persons and/or 40 households, depending on the census data topic. Conceptually, uniform grid squares may be the statistical ideal, but in practice it is often very difficult to accurately match source data with these units [77,78]. Most typically, in Canada, the 6-digit alphanumeric postal code is now available in disease registries and health encounter files. Exact physical locations (e.g. civic addresses) of principal residence and worksite are usually not captured in these databases, although they may be captured in other administrative files, such as municipal property files and national tax files. The ready availability of conversion software does facilitate the linkage of postal codes to physical coordinates, albeit not without some error, particularly in rural areas [26].

A concern often raised about the SIR is that two or more small areas may not be directly comparable since they are not based on the same standard population [79]. In practice, these comparisons will only be misleading if the age structure of these populations are extremely disparate [80]. Jarup and Best conclude that the imprecision of the directly standardized ratio is a far more serious problem [77].

Caution must be exercised with regards to the potential for and impact of over-stratification in disease mapping studies. For disease mapping models it is key to have reliable age- sex- and covariate-specific rates for the reference region, since these are in turn projected onto the area-specific populations also stratified by age, sex and covariates(s) and used to calculate expected values and SIRs. Adding relevant stratification variables further reduces the reference population in each cell. If the stratum-specific reference disease rates are unstable due to low counts this will generate unreliable expected values and standardized incidence ratios. To avoid problems of over stratification we recommend: 1) using a suitable time period for the analysis such that the size of the population in both the numerator and denominator are sufficient; 2) using a considerably large enough geographic area as a reference population for the same reasons as in point 1; and 3) using a small number of covariates that are known to have a significant impact on the disease in question. Users should still be aware that under certain conditions, such as with very rare diseases, instability may still arise due to over-stratification. It is evident that further research is needed to assess the minimum stratum-size to guarantee the reliability of these estimates.

Concern has also been raised that hierarchical Bayesian random effects models, particularly the commonly used BYM model, may over-smooth the variation in disease risk, particularly if the data are sparse [81,82]. That is, true clusters may have been smoothed away. Jarup urges caution in over-interpreting small area maps, whether smoothed or not, and whether the pattern shows a lack of spatial variation, or the opposite [77].

More recent work with simulation modelling has confirmed that the BYM model is conservative, with lower sensitivity at detecting areas with truly raised relative risks in the moderate range (i.e., 1.5-2.0). However, specificity is very high, even where data are sparse, reducing the risk of false alarms [23,46]. Richardson concludes that reasonable sensitivity (e.g., BYM model posterior probabilities of at least 70-80%) can be achieved for a range of cluster scenarios having SIRs in the moderate range (1.5-2.0) and moderate expected counts (~20). For areas with larger SIRs (~3.0), the likelihood of detection is high, even with small expected counts (~5), although the mean SIR is typically smoothed to half of the true value [23]. Thus, in our study, while it seems there was low sensitivity for detecting individual DAs of truly excess risk, the large size of the clusters of lung and prostate cancers we did detect, with 60 and 140 expected cases, respectively, suggests we had considerable study power to detect clusters of this size.

We believe that Moran's *I *and the spatial scan statistic (SaTScan), if used in conjunction with Bayesian smoothing, can strengthen spatial analysis. Moran's *I *provides a useful screen of the entire study area for spatial aggregation, and the spatial scan statistic is useful for identifying contiguous areas of statistically elevated risk, with supporting evidence from the Bayesian posterior distribution to help identify those individual areal units that contribute most strongly to the observed cluster [72]. Additionally, the Bayesian model is useful for providing more accurate, area-specific estimates of risk, for visualizing spatial patterns across the entire study area to create informative maps, and for estimating the effect of possible confounders on the spatial patterns [1-3,83,84].

In terms of the three goals for disease mapping using choropleth maps stated previously, our approach demonstrates the difficulty in finding a single optimal solution [83,84]. Clearly, we have been successful at producing more stable, accurate estimates of the underlying risk at the DA level, employing the BYM model. We have also been successful in detecting relatively large aggregations representing contiguous DAs where the risk of prostate cancer and male lung cancer are moderately high, in spite of the larger areal (WDG) risk estimates being lower than expected.

We acknowledge the sensitivity is quite low for detecting individual DAs with significantly higher, or lower, risks. Clearly, if the aim of our study was to estimate risk about a local point source of concern, then this disease-mapping approach is not optimal, and focused models that make use of additional information about proximity and/or exposure levels are required.

Finally, we recognize that our approach to assessing the shared spatial component of tobacco-associated cancers was not optimal. In the future, we will have to develop useful joint spatial and spatio-temporal Bayesian models to evaluate the similarity of spatial patterns, and better understand the long latencies and lags in relation to the etiology of most tumours [60,64,65].

Traditionally, maps of disease risk mostly show point estimates, without confidence intervals, displayed in the form of quantiles on choropleth maps [53]. Statements about uncertainty in these maps may be buried in footnotes or in the methods section. A possible solution, as we have provided here, is to map the posterior probabilities that an area exceeds a pre-specified threshold [41]. This may be presented on a separate map, or overlaid on the original choropleth map. It is quite apparent that sensitivity in detecting areas of higher, or lower, risk is substantially improved by exploiting the whole posterior distribution, rather than just mapping the mean values of the posterior distribution [23].

## Conclusions

This paper demonstrates the feasibility and utility of a standardized approach to identifying spatial clusters of neighbourhoods that have significantly higher risks of cancer while reducing noise in the small area estimates. This exploratory, ecologic study offers several hypotheses for the spatial patterns we identified within the area served by a Canadian public health unit. To the best of our knowledge, this is the first Canadian study published in the peer-reviewed literature estimating the risk of relatively rare public health outcomes at such a small areal level, namely the dissemination area.

While we restricted the use of the BYM Bayesian model to those cancers most likely to display evidence of spatial clustering (according to Moran's *I *statistic), we recognize that this mapping method is valuable for reducing noise in small area analysis, even in the absence of spatial autocorrelation. It provides useful smoothing or stabilizing of risk estimates, particularly where risks are extreme but highly uncertain, because of small observed and expected counts.

We believe the methods and tools needed to support small area analysis of public health outcomes and interventions are now readily available. However, data access and skillful spatial analysis may be important limitations for many public health units. Thus, a central public health infra-structure should be considered for necessary training and support, and for the construction and maintenance of a readily accessible integrated geodatabase, housing: relevant health outcomes; risk factors, interventions and other determinants of health; environmental hazards and exposures; and the requisite population and demographic data.

## Approvals

### Research Ethics

The research presented here was approved by Ontario Cancer Research Ethics Board on June 7, 2007 (OCREB #07-012).

### Privacy

This manuscript was reviewed by the Privacy Office at Cancer Care Ontario on February 25, 2010. In accordance with *Personal Health Information Protection Act (PHIPA) *legislation, to which Cancer Care Ontario is subject, the manuscript is in keeping with respect for personal privacy, safeguarding of confidential information, and the security of personal health information and thus does not possess any issues relating to privacy.

## Competing interests

The authors declare that they have no competing interests.

## Authors' contributions

EH, TN and SW contributed to the conception and design of the study, TN and SW performed the mapping and statistical analysis, JA and LB provided technical advice about mapping and spatial analysis, EH prepared the first draft and all authors contributed to the writing of the manuscript.

## Supplementary Material

Additional file 1 **Table 1 Cancer Sites**. 'Additional file 1 - Table 1: Descriptive Statistics and Moran's *I *for Cancer Sites Examined in Wellington-Dufferin-Guelph'. Click here for file

Additional file 2 **Appendix A - BYM model**. 'Additional file 2 - Appendix A: BYM Model in WinBUGS as employed by the RIF'. Annotated WinBUGS code for BYM model. Click here for file

Additional file 3 **Appendix B - Moran's I**. 'Additional file 3 - Appendix B: Parametric Bootstrap Derivation of Moran's *I *statistic'. *R *code for derivation of parametric bootstrap for Moran's *I *statistic. Click here for file

Additional file 4 **Table 2: WDG Characteristics**. 'Additional file 4 - Table 2: Characteristics of Wellington-Dufferin-Guelph'. Click here for file
