# Supplementary material for: Feasibility and utility of mapping disease risk at the neighbourhood level within a Canadian public health unit: an ecological study
Source: Int J Health Geogr. 2010 May 10;9:21. doi: 10.1186/1476-072X-9-21 (PMC2887786; doi:10.1186/1476-072X-9-21)
Supplement: Additional file 1 — Table 1 Cancer Sites. 'Additional file 1 - Table 1: Descriptive Statistics and Moran's I for Cancer Sites Examined in Wellington-Dufferin-Guelph'. [file 1476-072X-9-21-S1.PDF]

Additional file 1 - Table 1: Descriptive Statistics and Moran's *I* for Cancer Sites Examined in Wellington-Dufferin-Guelph

Cancer Site (ICD-9 code)*	Sex	Observed	Expected	SIR	SIR 95% CI	Median observed value◇	Range observed (min, max)◇	Bootstrap Moran's ***I***	
								I	p value† α=0.002
Prostate (185)	Male	735	759.07	0.97	0.90-1.04	2	0,22	**0.11**	**0.002**
Breast (174)	Female	702	699.33	1.00	0.93-1.08	2	0,3	0.02	0.296
Colorectal	Female	340	317.06	1.07	0.96-1.19	1	0,8	0.03	0.129
(153,154)	Male	331	363.47	0.91	0.82-1.02	1	0,6	0.01	0.348
Lung	Female	265	297.89	0.89	0.79-1.00	1	0,5	0.04	0.102
(162)	Male	337	397.53	0.85	0.76-0.94	1	0,7	**0.13**	**<0.001**
Melanoma	Female	104	78.30	1.33	1.10-1.61	0	0,3	-0.005	0.490
(172)	Male	107	93.68	1.14	0.95-1.38	0	0,3	0.08	0.010
Non-Hodgkin's Lymphoma	Female	91	107.75	0.84	0.68-1.04	0	0,3	-0.08	0.994
(200,202)	Male	95	130.86	0.73	0.59-0.89	0	0,3	0.03	0.155
Bladder	Female	39	45.56	0.86	0.61-1.17	0	0,2	0.04	0.078
(188)	Male	129	134.24	0.96	0.81-1.14	0	0,3	0.01	0.315
Leukemia	Female	66	62.49	1.06	0.82-1.34	0	0,2	0.02	0.203
(204, 205, 206, 207, 208)	Male	82	90.71	0.90	0.72-1.12	0	0,3	-0.03	0.799
Thyroid	Female	82	100.26	0.82	0.65-1.02	0	0,4	-0.07	0.987
(193)	Male	24	27.22	0.88	0.56-1.31	0	0,2	0.01	0.308
Ovarian (183)	Female	93	103.22	0.90	0.73-1.10	0	0,3	0.02	0.217
Acute Leukemia	Female	29	24.51	1.18	0.79-1.70	0	0,1	0.05	0.077
(204.0, 205.0, 206.0, 207.0, 208.0)	Male	37	32.54	1.14	0.80-1.57	0	0,3	-0.01	0.582
Testicular (186)	Male	39	33.40	1.17	0.83-1.60	0	0,2	0.02	0.258
Liver	Female	7	15.19	0.46	0.19-0.98	0	0,1	-0.01	0.464
(155)	Male	17	38.44	0.44	0.26-0.72	0	0,1	-0.02	0.654
Childhood leukemia	Female	9	4.84	1.86	0.85-3.53	0	0,1	-0.03	0.984
(204, 205, 206, 207, 208)	Male	6	6.80	0.88	0.32-1.92	0	0,1	-0.01	0.577
Smoking related sites exc. Lung	Female	313	324.17	0.97	0.86-1.08	1	0,7	0.01	0.321
(140-149, 150, 161, 188, 189)	Male	707	758.41	0.93	0.87-1.00	2	0.12	-0.01	0.577
Bladder & Kidney	Female	188	202.18	0.93	0.81-1.07	0	0,5	0.01	0.388
(188, 189)	Male	402	449.93	0.89	0.81-0.99	1	0,10	-0.04	0.871
Upper aero-digestive	Female	125	121.99	1.02	0.86-1.22	0	0,4	-0.02	0.658
(140-149, 150, 161)	Male	305	308.48	0.99	0.88-1.11	1	0,7	0.002	0.426

Significant Moran's *I*, after Bonferroni correction, displayed in **bold**
*0-89+ years examined for all cancers, except 0-14 year olds examined for childhood leukemia
◇among all dissemination areas in WDG
† one tailed test, adjusted for 24 multiple tests using Bonferroni correction, i.e., α<0.002.

Manuscript title: Feasibility and utility of mapping disease risk at the neighbourhood level within a Canadian public health unit: an ecological study. Holowaty *et al*.
