# Supplementary material for: Feasibility and utility of mapping disease risk at the neighbourhood level within a Canadian public health unit: an ecological study
Source: Int J Health Geogr. 2010 May 10;9:21. doi: 10.1186/1476-072X-9-21 (PMC2887786; doi:10.1186/1476-072X-9-21)
Supplement: Additional file 2 — Appendix A - BYM model. 'Additional file 2 - Appendix A: BYM Model in WinBUGS as employed by the RIF'. Annotated WinBUGS code for BYM model. [file 1476-072X-9-21-S2.PDF]

Additional file 2 - Appendix A: BYM Model in WinBUGS as employed by the RIF

Model
{
for(i in 1:N) *the number of areas[i] in the study*
{
Y[i] ~ dpois(lambda[i]) *the Poisson likelihood distribution for the observed count Y[i] in area[i] where lambda[i] is a function of the SIR[i] and the expected count, E[i]*

log(lambda[i]) <- log(E[i]) + alpha + a[i] + b[i]

smoothedSMR[i] <- exp(alpha + a[i] + b[i]) *the log of the smoothed SMR is a linear function of both fixed effects and random effects. In this case, alpha denotes the overall log of the SIR (i.e., for the entire study region), a[i] denotes unknown random effects that are not spatially correlated, and b[i] denotes random effects that ARE spatially correlated.*

It should be noted that where additional fixed covariates (e.g. neighbourhood income quintiles) are included in the model, these are subsumed in the E[i] term.

residRR[i] <- exp(a[i] + b[i]) *the residual relative risk is simply the RR of each area over(or under) the total study region's SMR.*

postprob[i] <- step(a[i]+b[i]) *the posterior probability for each area is simply the likelihood distribution for the SIR in each area [i].*

}

# Prior distributions

alpha ~ dflat() *this denotes a fixed effect, i.e., assumption that the overall SIR doesn't vary.*

overallRR <- exp(alpha) *the overall SIR across the entire study region is simply alpha exponentiated.*

for(i in 1:N){

a[i] ~ dnorm(0, tau.a) *prior distribution for the uncorrelated heterogeneity; assumption that the non-spatial random effects for each area[i] are distributed according to the normal distribution, with a standardized mean of zero and a variance of tau.a.*

}

b[1:N] ~ car.normal(adj[],weights[],num[],tau.b) *Conditionally autoregressive distribution for spatially correlated heterogeneity; assumption that the spatial random effects for each area[i] are conditional on adjacency for each area[i], and weighted according to the strength of the adjacency and with a variance of tau.b.*

# Hyperpriors

tau.a ~ dgamma(0.5, 0.0005) *These parameters of variability are also assumed to be variable and not fixed. Thus, tau.a is assumed to follow the gamma distribution, where the scale parameter (κ=0.5) and shape (θ =0.0005) parameters are fixed. This is usually a skewed distribution with mean=κ/θ and variance= κ / θ^2*

tau.b ~ dgamma(0.5, 0.0005) *Tau.b is also assumed to follow the gamma distribution, where the scale parameter and shape parameter are fixed.*

# Weights for the car.normal distribution

for(i in 1:sumNumNeigh){
weights[i] <- 1 *Where areas are adjacent, the assigned weight is one (1). Otherwise zero.*

}

# Variability of the RRs explained by the spatial factor

marginal.var.a <- sd(a[])*sd(a[])
marginal.var.b <- sd(b[])*sd(b[])
fracspatial <- marginal.var.b / (marginal.var.a + marginal.var.b) *This is a global estimate for the fraction of all variation in the SIRs attributable to the spatial effect.*
}
