# Supplementary material for: Feasibility and utility of mapping disease risk at the neighbourhood level within a Canadian public health unit: an ecological study
Source: Int J Health Geogr. 2010 May 10;9:21. doi: 10.1186/1476-072X-9-21 (PMC2887786; doi:10.1186/1476-072X-9-21)
Supplement: Additional file 3 — Appendix B - Moran's I. 'Additional file 3 - Appendix B: Parametric Bootstrap Derivation of Moran's I statistic'. R code for derivation of parametric bootstrap for Moran's I statistic. [file 1476-072X-9-21-S3.PDF]

Additional file 3 - Appendix B: Parametric Bootstrap Derivation of Moran's *I* statistic.

R Code

```
##########################
# read in the shapefiles; calculate adjacent neighbours and weights
##########################
library(maptools)
WDGPHU_LungM9903_nocov_DA2001.shp <- readShapePoly(
    fn="P:\\PopStudies\\OHEIS\\Studies\\WellingtonDufferinPHU\\Shapefiles\\WDGPHU_LungM9903_nocov_DA2001.shp",
    IDvar="DA2001")

##########################
# define neighbours and weights
##########################
library(spdep)
WDGPHU_DA2001.nb <- poly2nb(WDGPHU_Prost9903_nocov_DA2001.shp,
    WDGPHU_Prost9903_nocov_DA2001.shp$DA2001, queen=T) #constructs neighbours list
WDGPHU_DA2001.listw <- nb2listw(neighbours=WDGPHU_DA2001.nb, style="B", zero.policy=FALSE) #create weights
plot.listw(x=WDGPHU_DA2001.listw, coords=coordinates(WDGPHU_Prost9903_nocov_DA2001.shp), col="black", points=T,
    add=T, arrows=F) #plot neighbourhood definition

##########################
# bootstrap Moran's I from DCluster package
##########################
library(DCluster)
#####lung male
#create a data frame
WDGPHU_LungM9903_nocov_DA2001.ObsExp <-
    data.frame(Observed=WDGPHU_LungM9903_nocov_DA2001.shp$Observed)

#add Expected to data frame
WDGPHU_LungM9903_nocov_DA2001.ObsExp <- cbind(WDGPHU_LungM9903_nocov_DA2001.ObsExp,
    Expected=WDGPHU_LungM9903_nocov_DA2001.shp$Expected)
WDGPHU_LungM9903_nocov_DA2001.ObsExp.moran.pboot <- boot(WDGPHU_LungM9903_nocov_DA2001.ObsExp,
    statistic=moranI.pboot, sim="parametric", ran.gen=poisson.sim, R=999, listw=WDGPHU_DA2001.listw,
    n=length(WDGPHU_DA2001.nb), S0=Szero(WDGPHU_DA2001.listw) )
WDGPHU_LungM9903_nocov_DA2001.ObsExp.moran.pboot #t0 is Moran's I
plot(WDGPHU_LungM9903_nocov_DA2001.ObsExp.moran.pboot) #Display results
mean(abs(WDGPHU_LungM9903_nocov_DA2001.ObsExp.moran.pboot$t)>abs(WDGPHU_LungM9903_nocov_DA2001.Obs
    Exp.moran.pboot$t0)) #calculates p-value
```
