# Supplementary material for: Feasibility and utility of mapping disease risk at the neighbourhood level within a Canadian public health unit: an ecological study
Source: Int J Health Geogr. 2010 May 10;9:21. doi: 10.1186/1476-072X-9-21 (PMC2887786; doi:10.1186/1476-072X-9-21)
Supplement: Additional file 4 — Table 2: WDG Characteristics. 'Additional file 4 - Table 2: Characteristics of Wellington-Dufferin-Guelph'. [file 1476-072X-9-21-S4.PDF]

Additional file 4 - Table 2: Characteristics of Wellington-Dufferin-Guelph[1]

Characteristic	City of Guelph (in Wellington County)	Wellington County [2] (excluding Guelph)	Town of Orangeville (in Dufferin County)	Dufferin County [3] (excluding Orangeville)	Wellington-Dufferin-Guelph Public Health Unit	Province of Ontario
Area: km^2 (% WDG)	86.67 (2%)	2,569.99 (62%)	15.57 (<1%)	1,470.11 (35%)	4,142.33 (100%)	907,639.76
Population (% WDG)	106,170 (45%)	81,143 (34%)	25,248 (11%)	25,765 (11%)	238,326 (100%)	11,410,046
Average Population Density: persons/km^2 (range for DA)	1,225.06 (62.14 – 2,315,000)	31.57 (0-1,110,000)	1,621.50 (0-2,040,000)	17.53 (5.79-950,000)	57.53 (0-2,315,000)	12.57 (0-13,980,000)
Dissemination Areas (DA) (% WDG)	166 (50%)	102 (31%)	27 (8%)	36 (11%)	331 (100%)	18,584
Average DA population (range)	639.58 (229-3,167)	795.52 (0-1,862)	935.11 (0-1,612)	715.69 (34-1,236)	720.02 (0-3,167)	613.97 (0-11,657)
Census Tracts (CT) (% WDG)	21 (75%)	2 (7%)	4 (14%)	1 (4%)	28 (100%)	2,013
Average CT population (range)	5,055.71 (1,610-9,373)	5,587 (5,053-6,121)	6,312 (5,273-7,958)	6,922 (6,922-6,922)	5,339.79 (1,610-9,373)	4,558.73 (0-20,635)
Average Household Income Quintile (QAIPPE) [4] (% of area population)						
0 (suppressed) [5]	1	1	1	1	1	2
1 (low income)	22	3	8	4	12	19
2	22	18	47	8	22	19
3	22	12	30	13	18	20
4	13	26	14	31	19	20
5 (high income)	20	40	0	43	27	20

[1]Estimates from the 2001 Census of Canada
[2]Wellington County (CD) contains the CMA of Guelph as well as the CSD of the City of Guelph
[3]Dufferin County (CD) contains the CSD of Orangeville, which is part of the Toronto CMA
[4]QAIPPE is a measure of household income developed by Statistics Canada, indicating the quintile of average annual household income per DA based on per person equivalents [30] relative to the Toronto CMA for the Town of Orangeville, the Guelph CMA for the City of Guelph and relative to the remainder of the province of Ontario for the rest of Wellington-Dufferin-Guelph.
[5]Average Household Income Quintile is suppressed in DAs in the lowest and highest quintiles where there are fewer than 40 private households or the population is less than 250.

NOTE: There are 8 DAs in Wellingon-Dufferin-Guelph for which age and sex group data are suppressed due to population counts less than 40 persons. These DAs are not included in the analysis.

Manuscript title: Feasibility and utility of mapping disease risk at the neighbourhood level within a Canadian public health unit: an ecological study. Holowaty *et al*.
